# Supplementary material for: PROVE—Pre-Eclampsia Obstetric Adverse Events: Establishment of a Biobank and Database for Pre-Eclampsia
Source: Cells. 2021 Apr 20;10(4):959. doi: 10.3390/cells10040959 (PMC8074755; doi:10.3390/cells10040959)
Supplement: Supplementary file 1 [file cells-10-00959-s001.zip › cells-1170206-supplementary.pdf]

Supplementary material

Supplementary material to:
Bergman L, Bergman K, Langenegger E, Moodley A, Griffith-Richards S, Wikström S, Hall D, Joubert L, Herbst P, Schell S, van Veen T, Belfort M, Tong S, Walker S, Hastie R, Cluver C. PROVE – Pre-eclampsia Obstetric Adverse Effects: Establishment of a biobank and database for pre-eclampsia.

Contents:

Supplementary Methods 2
- Cognitive function tests 2
- Brain magnetic resonance imaging (MRI) 2
  - Imaging sequences 2
  - Evaluation 3
- Transcranial Doppler 3
- Echocardiograms 4
- Cardiac MRI 5
- List of abbreviations 6
- References 8

Supplementary Methods

Cognitive function tests

The cognitive function questionnaire (CFQ) is a subjective questionnaire that assesses the likelihood of committing errors in the completion of daily tasks such as the routines of everyday life.(1) The CFQ consists of 25 items that are scored on a 5-point scale (range, 0 [never] to 4 [very often]). Thus, the total score ranges from 0-100, with higher scores indicating more frequently occurring cognitive failures.(1)

The Montreal Cognitive Assessment (MoCA) is an objective assessment composed of a variety of cognitive domains assessing attention, concentration, executive function, memory, language, visio-spatial abilities, abstract thinking, mathematical calculations and orientation.(2) The highest possible score is 30 points and a score of 26 points or greater indicates normal function in individuals with a total school education of more than 12 years. If total school education is less than 12 years, an additional point is added to the total score.(2)

These assessments are administered by trained research midwives. The CFQ test is available in English and Afrikaans and questions have been translated to Xhosa by an interpreter. The MoCA test is available in English and Afrikaans and performed in Xhosa if needed at time of assessment by the Xhosa speaking research midwife due to the lack of validated translations.

Brain magnetic resonance imaging (MRI)

Imaging sequences

- Sagittal 2D T1 weighted spin echo sequence with repetition time (TR)/echo time (TE)/flip angle (FA)/ nr of excitations (NEX): 389 ms/ 9.7 ms/ 150°/ 3; acquired voxel size: 0.6 mm x 0.7 mm x 4.0 mm, and acquisition time 2:30 min.
- Axial T2 weighted spin echo sequence with TR/TE/FA/NEX: 5720 ms/ 80 ms/ 150°/ 3; acquired voxel size: 0.6 mm x 0.6 mm x 4.0 mm, and acquisition time 2:46 min.
- Axial 2D Fluid attenuated inversion recovery (FLAIR) sequence with TR/TE/FA/Inversion time/NEX: 8000 ms/ 84 ms/ 150° / 2370 ms/ 1; acquired voxel size: 0.7 mm x 1.0 mm x 4.0 mm, and acquisition time 2:40 min.
- Diffusion weighted imaging (DWI) sequence with TR/TE/FA/NEX: 6500 ms/ 119 ms/ 90°/1; acquired voxel size: 1.2 mm x 1.2 mm x 3.0 mm, acquisition time 3:04 min, and b values of 0, 50, 100, 150, 200, 400, 600, 800 and 1000 $x10^{-3}$ s/mm^2.

- 3D TOF Magnetic resonance angiography (MRA) sequence with TR/TE/FA/NEX: 27 ms/ 7 ms/ 25° / 1; acquired voxel size: 0.7 mm x 0.7 mm x 0.5 mm, and acquisition time 5:10 min.
- Axial T2* weighted gradient echo (GRE) sequence with TR/TE/FA/NEX: 830 ms/ 25 ms/ 20°/1; acquired voxel size: 0.9 mm x 0.9 mm x 4.0 mm, and acquisition time 3:31 min.

Evaluation

T1-, T2-, T2*-weighted, and FLAIR sequences are used for morphological assessment, including detection of oedema, hemorrhages and other parenchymal changes. Location of changes is noted, and the volume of oedema is measured.
MRA images are used for assessment of stenoses in intracranial arteries. Arterial stenoses are assessed in the A1, A2, M1, P1, and P2 segments; and graded into 0, 1-49%, 50-99%, and 100% stenoses.
High b-value DWI images are used for identification of acute infarcts. In addition, the DWI sequence with multiple b-values permits calculation of the following intravoxel incoherent motion imaging (IVIM) metrics: true diffusion (D), pseudodiffusion (D*), and perfusion fraction (f). Parametric maps of these metrics enable assessments of extracellular oedema (D), capillary blood flow (f x D*), and capillary blood volume (f). Measurements are made in predefined locations: bilateral frontal and parietooccipital white substance, bilateral lentiform and caudate nuclei, bilateral thalami, and in regions with oedema visualized on FLAIR images.

Transcranial Doppler

Transcranial Doppler (TCD) examinations are performed bedside during a single 7-minute episode. At the time of the TCD examination, brachial systolic (SBP) and diastolic (DBP) blood pressure are measured. Patients are studied in a supine or semi-Fowlers position in their hospital bed. Simultaneous TCD evaluation of both middle cerebral arteries (MCA) is carried out using 2 MHz pulsed, range gated transcranial Doppler probes (Spencer Technologies, Seattle, WA), held in place using a head frame. If only one MCA can be found, that one side is used in the analysis. The depth of insonation is set at 45 to 65 mm with slight anterior angulation (15-30 degrees) of the probe through the temporal window. The MCA is identified using M-mode to detect the MCA/anterior cerebral artery (ACA) bifurcation, the expected velocity and the depth.
Blood pressure is continuously measured non-invasively using finger arterial volume clamping (Finometer Pro, Finapres Medical Systems, Amsterdam, The Netherlands) with the servo-adjust switched off. This is subsequently calibrated with the brachial blood pressure (BP). The BP tracing also serves to mark each cardiac cycle. End-tidal

CO_2 ($EtCO_2$) is measured with a nasal cannula (Nellcor Oximax N-85, Covidien, Mansfield, MA).

All data are recorded at 50 Hz, interpolated to 200 Hz and visually inspected during analysis to remove occasional large spikes. A median filter is used to remove small spikes and artifacts in the cerebral blood flow velocity (CBFV) signal. All signals are then low-pass filtered with a Butterworth filter with a cutoff frequency of 20 Hz. Mean BP, bilateral CBFV, $EtCO_2$ and heart rate are then calculated for each beat. The critical closing pressure (CrCP) and resistance-area product (RAP) are obtained using the first harmonic of BP and CBFV of each cardiac cycle.(3) All signals are then resampled at 5 Hz.

Cerebral autoregulation is determined from the CBFV responses to spontaneous fluctuations in mean arterial BP as described previously.(4) Segments consisting of 512 samples and 50% superposition, are transformed with the fast Fourier transform (FFT) algorithm (Welch method), to obtain the transfer function parameters coherence, gain and phase in the low frequency range (<0.1 Hz). The inverse FFT is then performed to estimate the impulse and step responses. The CBFV step response to a sudden change in BP is compared to 10 template curves proposed by Tiecks *et al*.(4, 5) and the best fit curve corresponds to the autoregulation index (ARI).(4, 5) A value of ARI=9 represents the best observed cerebral autoregulation, while ARI=0 corresponds to absence of dynamic cerebral autoregulation (DCA). Measurements were rejected if mean coherence function was < 0.19 and when the normalized mean square error for fitting the step response was >0.30.(5) Baseline cerebral hemodynamic parameters are reported as the average over the 7 minute baseline recording.

Pulsatility (PI) and resistance (RI) indices and cerebral perfusion pressure (CPP) are calculated using the averages of the velocity and maternal blood pressure data as follows: PI=(Peak Systolic Velocity-Diastolic Velocity)/Mean Velocity, RI=(Peak Systolic Velocity-Diastolic Velocity)/Peak Systolic Velocity and CPP=[Mean Velocity/(Mean Velocity-Diastolic Velocity)](Mean Arterial Pressure-Diastolic Blood Pressure).(6)

Echocardiograms

The echocardiograms are performed using the General Electric Vivid 5S with tissue doppler imaging capabilities, in accordance with the European Society of Cardiology guidelines.(7)

The following standard echocardiographic measurements are performed: Left ventricle (LV) end-diastolic dimension (LVEDd), LV end-systolic dimension (LVESd), end-diastolic wall thickness of the interventricular septum (IVSd) and left ventricular posterior wall (PWTd). The LV mass is calculated according to the

American Society of Echocardiography (ASE) formula. The LV mass index (LVMI) is calculated by the indexation of LV mass to body surface are (BSA). The relative wall thickness (RWT) is calculated according to formula: RWT= (IVS + PWT)/LVDd, to classify the LV hypertrophy (LVH) and geometric pattern (concentric LVH: RWT>0.45; eccentric LVH: RWT<0.45).

LV systolic function is assessed with the following parameters: Fractional shortening; LV ejection fraction (Simpsons biplane method of disks); Sa wave tissue doppler imaging (TDI), myocardial performance index (MPI) and speckle tracking derived longitudinal and global systolic strain (GLS). Circumferential LV wall end systolic stress is measured and mid wall fractional shortening corrected for afterload is calculated as an index of LV muscle function independent of afterload.

LV diastolic function is assessed with the following parameters: Velocities of early trans mitral diastolic flow velocity (E) and flow velocity during atrial contraction (A); E/A ratio; E-wave deceleration time; isovolumetric relaxation time (IVRT); pulsed Doppler TDI at septal and lateral mitral annulus (E'); E/E' ratio; pulmonary vein pulsed wave Doppler; left atrial end systolic diameter (LAD es) in a parasternal long-axis view and maximal left atrial area (LAArea es) at end-systole in a four-chamber view.(8)

Cardiac MRI

Cardiac MRI is performed in accordance with current guideline recommendations.(9-11) A 1.5T cardiac MRI (Siemens Aera) is performed at enrolment or as soon as cardiac MRI can be tolerated. Cases have gadolinium (Gadovist© at a recommended dose of 0,2ml/kg) administered during the cardiac MRI. Controls have a baseline cardiac MRI at enrolment, but gadolinium is not administered. Analysis of the CMRI data is performed using CVI 42 (Circle cardiovascular imaging inc. Canada). Three long axis cine images are acquired in the two, four and three chamber orientation as well as short axis cine images using a breath-held retrospectively gated balanced steady state free precession (SSFP) gradient echo sequence. The slice thickness of these short axis stacks is 8mm at 10mm intervals (25% gap) from LV base to apex. The endocardial and epicardial contours are traced in short axis in end diastole and end systole to determine LV volume, mass and functional parameters. Papillary muscles are excluded from the blood pool and contribute to myocardial mass. All volumes and masses are indexed to body surface area as calculated using the DuBois formula (0.0247 X height (m)$^{0.725}$ X weight (kg)$^{0.425}$).

Oedema imaging is performed using short-tau inversion recovery (STIR) sequences. A basal mid and apical short axis image is acquired. In post processing, The LV is segmented into the 16 segment AHA model.(12) A polar map of raw STIR values is generated using endo and epicardial offset of 20% to ensure only mypocardium is included. These are then compared to the signal intensity value of serratus anterior

on the same slice and a polar map of cardiac muscle to skeletal muscle signal intensity ratios can be extrapolated for each patient. This semi automated method reduces operator bias and allows for more detailed description of cardiac oedema. T1 and T2 native imaging is acquired using the shortened modified look locker technique (shMOLLI). 3 short axis images are acquired at the level of the basal, mid and apical LV used for quantification. Four, two and three chamber images are also acquired for cross correlation. Similar to STIR images, Polar maps of Native T1 and T2 values are generated by segmenting the LV into 16 AHA segments, using 20% epi and endocardial offset. This allows for objective assessment of both segmental native T1 and T2 values as well as global, basal, mid and apical LV T1 and T2 values.

List of abbreviations

ACA; Anterior Cerebral Artery
ASE; American Society of Echocardiography
ARI; Autoregulation Index
BP; Blood Pressure
BSA; Body Surface Area
CBFV; Cerebral Blood Flow Velocity
CFQ; Cognitive Failure Questionnaire
CPP; Cerebral Perfusion Pressure
CrCP; Critical Closing Pressure
DBP; Diastolic Blood Pressure
DWI; Diffusion Weighted Imaging
DCA; Dynamic Cerebral Autoregulation
$EtCO_2$; End-tidal CO_2
FA; Flip Angle
FFT; Fast Fourier Transform
FLAIR; Fluid Attenuated Inversion Recovery
GRE; Gradient Echo
IVIM; Intravoxel Incoherent Motion
IVSd; end-diastolic wall thickness of the interventricular septum
LAArea es; Maximal left atrial area
LAD es; Left atrial end systolic diameter
LV; Left Ventricle
LVEDd; Left Ventricle End Diastolic Dimension
LVESd; Left Ventricle End Systolic Dimension
LVH; Left Ventricular Hypertrophy
LVMI; Left Ventricular Mass Index
MCA; Middle Cerebral Artery
MoCA; Montral Cognitive Assessment
MPI; Pyocardial Performance Index
MRA; Magnetic Resonance Angiography

MRI; Magnetic Resonance Imaging
NEX; Number of Excitations
PI; Pulsatility index
PWTd; end-diastolic wall thickness of the left ventricular posterior wall
RAP; Resistance Are Product
RI; Resistance Index
RWT; Relative Wall Thickness
TCD; Transcranial Doppler
TDI; Tissue Doppler Imaging
TE; Echo Time
TR; Repitition Time
SBP; Systolic Blood Pressure
shMOLLI; Shortened Modified Look Locker Technique
STIR; Short-Tau Inversion Recovery

References

1. Broadbent DE, Cooper PF, FitzGerald P, Parkes KR. The Cognitive Failures Questionnaire (CFQ) and its correlates. Br J Clin Psychol. 1982;21 (Pt 1):1-16.
2. Nasreddine ZS, Phillips NA, Bedirian V, Charbonneau S, Whitehead V, Collin I, et al. The Montreal Cognitive Assessment, MoCA: a brief screening tool for mild cognitive impairment. J Am Geriatr Soc. 2005;53(4):695-9.
3. Panerai RB, Salinet AS, Brodie FG, Robinson TG. The influence of calculation method on estimates of cerebral critical closing pressure. Physiol Meas. 2011;32(4):467-82.
4. Panerai RB, White RP, Markus HS, Evans DH. Grading of cerebral dynamic autoregulation from spontaneous fluctuations in arterial blood pressure. Stroke; a journal of cerebral circulation. 1998;29(11):2341-6.
5. Tiecks FP, Lam AM, Aaslid R, Newell DW. Comparison of static and dynamic cerebral autoregulation measurements. Stroke; a journal of cerebral circulation. 1995;26(6):1014-9.
6. Belfort MA, Tooke-Miller C, Varner M, Saade G, Grunewald C, Nisell H, et al. Evaluation of a noninvasive transcranial Doppler and blood pressure-based method for the assessment of cerebral perfusion pressure in pregnant women. Hypertension in pregnancy : official journal of the International Society for the Study of Hypertension in Pregnancy. 2000;19(3):331-40.
7. Lang RM, Badano LP, Mor-Avi V, Afilalo J, Armstrong A, Ernande L, et al. Recommendations for cardiac chamber quantification by echocardiography in adults: an update from the American Society of Echocardiography and the European Association of Cardiovascular Imaging. Eur Heart J Cardiovasc Imaging. 2015;16(3):233-70.
8. Nagueh SF, Smiseth OA, Appleton CP, Byrd BF, 3rd, Dokainish H, Edvardsen T, et al. Recommendations for the Evaluation of Left Ventricular Diastolic Function by Echocardiography: An Update from the American Society of Echocardiography and the European Association of Cardiovascular Imaging. Eur Heart J Cardiovasc Imaging. 2016;17(12):1321-60.
9. Moon JC, Messroghli DR, Kellman P, Piechnik SK, Robson MD, Ugander M, et al. Myocardial T1 mapping and extracellular volume quantification: a Society for Cardiovascular Magnetic Resonance (SCMR) and CMR Working Group of the European Society of Cardiology consensus statement. J Cardiovasc Magn Reson. 2013;15:92.
10. Friedrich MG, Sechtem U, Schulz-Menger J, Holmvang G, Alakija P, Cooper LT, et al. Cardiovascular magnetic resonance in myocarditis: A JACC White Paper. Journal of the American College of Cardiology. 2009;53(17):1475-87.
11. Ferreira VM, Schulz-Menger J, Holmvang G, Kramer CM, Carbone I, Sechtem U, et al. Cardiovascular Magnetic Resonance in Nonischemic Myocardial Inflammation: Expert Recommendations. Journal of the American College of Cardiology. 2018;72(24):3158-76.

12. Cerqueira MD, Weissman NJ, Dilsizian V, Jacobs AK, Kaul S, Laskey WK, et al. Standardized myocardial segmentation and nomenclature for tomographic imaging of the heart. A statement for healthcare professionals from the Cardiac Imaging Committee of the Council on Clinical Cardiology of the American Heart Association. Circulation. 2002;105(4):539-42.
